# Myristic acid alleviates hippocampal aging correlated with GABAergic signaling

**DOI:** 10.3389/fnut.2022.907526

**Published:** 2022-09-08

**Authors:** Qi Shang, Guifeng Chen, Peng Zhang, Wenhua Zhao, Honglin Chen, Die Yu, Fuyong Yu, Huiwen Liu, Xuelai Zhang, Jiahui He, Xiang Yu, Zhida Zhang, Riwei Tan, Zixian Wu, Jingjing Tang, De Liang, Gengyang Shen, Xiaobing Jiang, Hui Ren

**Affiliations:** ^1^Guangzhou University of Chinese Medicine, Guangzhou, China; ^2^Lingnan Medical Research Center of Guangzhou University of Chinese Medicine, Guangzhou, China; ^3^The First Affiliated Hospital of Guangzhou University of Chinese Medicine, Guangzhou, China; ^4^Nanshan Hospital, The First Affiliated Hospital of Guangzhou University of Chinese Medicine (Shenzhen Nanshan Hospital of Chinese Medicine), Guangzhou, China

**Keywords:** myristic acid, hippocampus, aging, GABAergic expressions, GABRB2, GABRA2

## Abstract

Previous studies have shown that myristic acid (MA), a saturated fatty acid, could promote the proliferation and differentiation of neural stem cells *in vitro*. However, the effect of MA on hippocampal neurons aging has not been reported *in vivo*. Here we employed 22-month-old naturally aged C57BL/6 mice to evaluate the effect and mechanism of MA on hippocampal aging. First, we examined a decreased exploration and spatial memory ability in aging mice using the open field test and Morris water maze. Consistently, aging mice showed degenerative hippocampal histomorphology by H&E and Nissl staining. In terms of mechanism, imbalance of GABRB2 and GABRA2 expression in aging mice might be involved in hippocampus aging by mRNA high throughput sequencing (mRNA-seq) and immunohistochemistry (IHC) validation. Then, we revealed that MA alleviated the damage of exploration and spatial memory ability and ameliorated degeneration and aging of hippocampal neurons. Meanwhile, MA downregulated GABRB2 and upregulated GABRA2 expression, indicating MA might alleviate hippocampal aging correlated with GABAergic signaling. In conclusion, our findings revealed MA alleviated hippocampal aging correlated with GABAergic signaling, which might provide insight into the treatment of aging-associated diseases.

## Introduction

With the rapid increase in the global aging population, aging-related diseases have brought a huge burden to patients, families, and socioeconomics. It is imminent to explore the pathogenesis and therapeutic drugs of aging. The hippocampus is an important part of the limbic system of the brain and contains various messenger receptors (1). It is not only a high-level regulatory center of neuroendocrine system but also the most sensitive area (1, 2). Studies have confirmed that hippocampus function is closely related to aging (3, 4). The decline of hippocampal neurons is the most pronounced change with age (5). These changes in hippocampus are closely related to age-related exploration and spatial memory impairment as well as an imbalance of hippocampal messenger receptors (6, 7). Therefore, a more in-depth understanding of hippocampus aging is essential for the improvement of treatment and prevention of aging-associated diseases.

GABAergic neural circuits play an important role in regulating hippocampal neuronal synaptic plasticity, memory, and cognitive function (8). GABAergic interneurons synthesize and release the neurotransmitter γ-aminobutyric acid (GABA) and contribute substantially to the regulation of the adult neuronal network (8). Generally, GABA exerts its effects on nerves mainly through its receptors; GABRA2 (GABAA receptor subunit alpha2) and GABRB2 (GABAA receptor subunit beta2) are the two important subunits of GABAA receptor, respectively. Previous studies demonstrated that GABRA2 expression decreased with age in the hippocampus (9, 10). However, aging-related disorders on GABRB2 expression are contradictory and need further experimental validation.

Previous studies have shown that myristic acid (hereinafter referred to as MA, IUPAC name: tetradecanoic acid, molecular formula: CH_3_(CH_2_)_12_COOH, CAS#: 544-63-8), a saturated fatty acid, mainly present in cardamom oil, coconut oil, palm kernel oil, butterfat, and bovine milk, could be used to prepare a variety of foods (11, 12). MA could promote the proliferation and differentiation of neural stem cells *in vitro* (13). Besides, aging was accompanied by a decline in MA, indicating MA played an important role in aging-related disorders (14). The isomer of MA, Myristoleic acid, could suppress osteoclastogenesis and bone resorption by inhibiting RANKL activation, indicating MA had a potential therapeutic effect on bone aging-related diseases, such as osteoporosis and osteoarthritis (15). However, the effect of MA on hippocampal neurons aging has not been reported *in vivo*. Here we employed 22-month-old naturally aged C57BL/6 mice to evaluate the effect and mechanism of MA on hippocampal aging. Our investigations indicated that MA alleviated hippocampal aging correlated with GABAergic signaling, which may provide insight into the treatment of aging-associated diseases.

## Materials and methods

### Mice

C57BL/6 male mice aged 3 and 22 months were obtained from the Laboratory Animal Center of Guangzhou University of Chinese Medicine (GZUCM) (Animal production certificate#(Yue)20180034). Mice had *ad libitum* access to tap water and standard rodent chow (Product performance standard: GB14924.3-2010, Laboratory Animal Center of GZUCM). All animal experiments were approved by the Ethics Committee of the First Affiliated Hospital of GZUCM (approval no. TCMF1-2021026). Firstly, the mice were divided into two groups: 3-month-old mice (3M) and 22-month-old mice (22M) group to assess the success of natural aging murine models. Secondly, another batch of 22-month-old mice was randomly divided into two groups: 22-month-old mice (22M, equal amount of solvent intraperitoneal injection) and MA (MA+22M, 2mg/kg·d MA intraperitoneal injection) group. They were randomized to each group after matching for age and body mass. Drugs were administered for 2 months by intraperitoneal injection. The concentration of MA was referred to a previous study (15). Following the behavioral assessment (open field test and Morris water maze), samples from murine brains were used for subsequent experimental assessments.

### Open field test

The operation of open field test process was in accordance with a previous study which was used for assessing murine exploratory behavior (16). Generally, the exploration ability of aging mice decreased (17). Briefly, mice were transported to the laboratory room 2 h in advance to adapt to the environment before the experiment. The open field reaction box for C57BL/6 was a 50 × 50 cm open field chamber. The open-field chamber was divided into nine grids. The central area is defined as the center 1/9 area of open-field chamber bottom, and the remaining area is defined as the peripheral area. The longer time the mice spent in the center, longer distance the mice across the center and more times the mice across the center in the open-field chamber, the better the mouse's ability to explore (17). Wipe the bottom of open-field chamber with 75% alcohol. Gently remove the animals from the cage, place them quickly in the open-field chamber. Set up the parameters in SuperMaze software (Version 3.0, Shanghai XinRuan Information Technology Co., Ltd) and automatically record the animal activities in the box for 5 min. After one mouse was finished, the bottom of the box was wiped with 75% alcohol to remove the odor, and the next mouse was tested with the same method before. The parameters including total distance traveled by the mice (Total Distance), mice quiescent time (Quiescent Time), distance in the central region (Distance Center), number of central region entries (Number of Center), central region staying time (Time Center), distance in the periphery region (Distance Periphery), number of periphery region entries (Number of Periphery), Periphery region staying time (Time Periphery), the ratio of Distance Center vs. Distance Periphery and Time Center vs. Time Periphery were evaluated.

### Morris water maze

Morris water maze was employed to assess the mouse spatial memory (6). After the open field test, the process of the Morris water maze was operated next day. Mice were placed into the pool from four quadrants facing the pool wall, and the time required by the mice from entering the water to finding and standing on the underwater concealed platform was recorded as the escape latency with SuperMaze software (Version 3.0, Shanghai XinRuan Information Technology Co., Ltd). If the mice could not find the platform 60 s after entering the water, the mice were guided to stay on the platform for 10 s. This was a positioning navigation test of mice, which was four quadrants a day for a total of four days. On the fifth day, a space exploration test was conducted. The platform was removed and the mice were placed from the opposite quadrant of the platform. The swimming time of the mice lasted 60 s. Each mouse was put into the pool from four water entry points for one training, and the interval between the two training was at least 30 min. The quadrants of each day's training were randomly arranged. The parameters including Escape Latency and Platform crossover number were recorded.

### HE staining and Nissl staining

The murine cerebral hemispheres and organs (Heart, liver, spleen, lung, kidney and testis) were removed completely on ice, fixed with 4% paraformaldehyde for 48 h, then embedded in paraffin. The hemispheres were cut to the hippocampus zone at the midsagittal plane of 5 μm, and stained with H&E staining solution (Servicebio, G1005, Wuhan) and Nissl staining solution (Servicebio, G1036, Wuhan) respectively. The organs including heart, liver, spleen, lung, kidney and testis were cut into 5 μm slices, and stained with H&E staining solution (Servicebio, G1005, Wuhan). The H&E and Nissl staining of sections were intelligently photographed by Digital Pathology Scanning System (3D HISTEC Ltd., Pannoramic MIDI). The rate of Nissl-stained cells and neurodegeneration of hippocampus were calculated using Image J 1.51 analysis software (Wayne Rasband, National Institutes of Health, USA). The rate of Neurodegeneration was defined as the ratio of pathological neuronal cells to total cells in the entire hippocampus; Neuronal pathological changes included disarrangement of neurons, nuclear shrinkage, and dark staining of neuronal cells (18). Each group had three biological replicates; each mouse was randomly selected two non-overlapping visual fields in each partial of hippocampal CA1, CA3 and DG area under high magnification microscope (400×) of each slice, and counted the mean rate of Nissl-stained cells and neurodegeneration.

### mRNA-seq of hippocampus

The total RNA was extracted from young (3M) and aging (22M) hippocampus samples in mice by using Trizol reagents (Invitrogen). The extracted total RNA samples were subjected to agarose electrophoresis and Nanodrop for quality inspection and quantification. Oligo magnetic beads were used to enrich the mRNA, and the KAPA Stranded RNA-Seq Library Prep Kit (Illumina, Aksomics, Shanghai) was used to construct the library. The constructed library was checked by Agilent 2100 Bioanalyzer (Aksomics, Shanghai), and the final quantification of the library was performed by qPCR. According to the quantitative results and the final sequencing data, the sequencing libraries of different samples were mixed into the sequencing process. The screening thresholds to determine the significant differential expression (DE) genes were *P* <0.05 and |log2 fold change (FC)| > 0.585 (19). The normalized data of each group were analyzed by hierarchical clustering. The color green represented low expression, whereas red represented high expression, to investigate the possible mechanism of the upregulated and downregulated DE genes involved in the process of hippocampal aging. The raw data of mRNA-seq of hippocampus were uploaded to GEO database (https://www.ncbi.nlm.nih.gov/geo/query/acc.cgi?acc=GSE201029).

### KEGG analysis

The differentially mRNAs expressions from mRNA-seq data were employed for KEGG analysis by using the KEGG database. The upregulated and downregulated mRNAs were uploaded into the KEGG database. The bar plot showed the top in front enrichment score value of the significant enrichment pathway in terms of the value of -log10 (*P*-value).

### Immumohistochemical staining (IHC)

IHC was used to detect the GABRB2, GABRA2, P16, and P21 protein expression in the brain. The brain tissue sections were dewaxed, antigen repaired, and sealed successively. GABRB2 (Bioss Cat# BS-12065R, Rabbit, 1:200), GABRA2 (Affinity Biosciences Cat# DF6626, RRID: AB_2838588, Rabbit, 1:100), P16 (Affinity Biosciences Cat# AF0228, RRID: AB_2833403, Rabbit, 1:200) and P21 (Beyotime Cat#AP021, 1:200) antibodies were added respectively and incubated at 4°C overnight. Then Goat anti-rabbit or mouse IgG antibodies were added on brain tissue sections and incubated at room temperature for 1 h. After conventional DAB staining, the sections were stained with hematoxylin and sealed. IHC of brain sections for the whole film scanning was intelligently photographed by Digital Pathology Scanning System (3D HISTEC Ltd., Pannoramic MIDI). Image J 1.51 analysis software (Wayne Rasband, National Institutes of Health, USA) was used to calculate the relative protein expressions. Each group had three biological replicates; each mouse was randomly selected two non-overlapping visual fields in each partial of hippocampal CA1, CA3 and DG area under high magnification microscope (400×) of each slice, and counted the mean optical density, and then normalized by the folds of experimental group to control group.

### Immunofluorescence (IF)

The paraffin sections of hippocampal tissue were deparaffinized with xylene, anhydrous ethanol, 85% alcohol and 75% alcohol, respectively. The tissue sections were placed in citric acid buffer (pH 6.0) in a microwave oven for antigen retrieval. Then the primary antibodies including GABRB2 (Bioss Cat# BS-12065R, Rabbit, 1:200), GABRA2 (Affinity Biosciences Cat# DF6626, RRID: AB_2838588, Rabbit, 1:100), Sox2 (Bioss Cat#BS-0523R, 1:200), Nestin (Proteintech Cat#19483-1-AP, 1:100) were placed in a humid box and incubated overnight at 4°C. Next, the secondary antibody Alexa Fluor 488 AffiniPure Goat Anti-Rabbit IgG (H+L) (Jackson Cat#111-545-003, 1:200) was incubated at room temperature for 50 min in the dark. Nuclei were counterstained with DAPI (Solarbio, C0060, 1:100) and incubated for 10 min at room temperature in the dark. After tissue sections drying, the sections were mounted with anti-fluorescence quenching mounting medium. IF of hippocampus sections for the whole film scanning was intelligently photographed by Digital Pathology Scanning System (3D HISTEC Ltd., Pannoramic MIDI). Image J 1.51 analysis software (Wayne Rasband, National Institutes of Health, USA) was used to calculate the relative protein expressions. Each group had three biological replicates; each mouse was randomly selected two non-overlapping visual fields in the hippocampal CA3 area under high magnification microscope (400×) of each slice, and counted the average fluorescence intensity, and then normalized by the folds of experimental group to control group.

### Statistical analysis

All data were checked for normality and homogeneity of variance. If two independent sample data fit a normal distribution, the results are expressed as means ± standard deviations (SDs) and two-tailed Student's *t*-test was used. If two independent sample data do not conform to a normal distribution, the Mann-Whitney U test was employed and a box plot was used. Two-way analysis of variance (ANOVA) following Bonferroni's multiple comparisons test was employed to analyze the Morris water maze data. *P* < 0.05 was considered statistically different. Statistical analysis was performed using GraphPad Prism version 8.4.3 for Windows (GraphPad Software, San Diego, California, USA).

## Results

### Aging mice displayed decreased exploration and spatial memory ability

**Figure 1:**
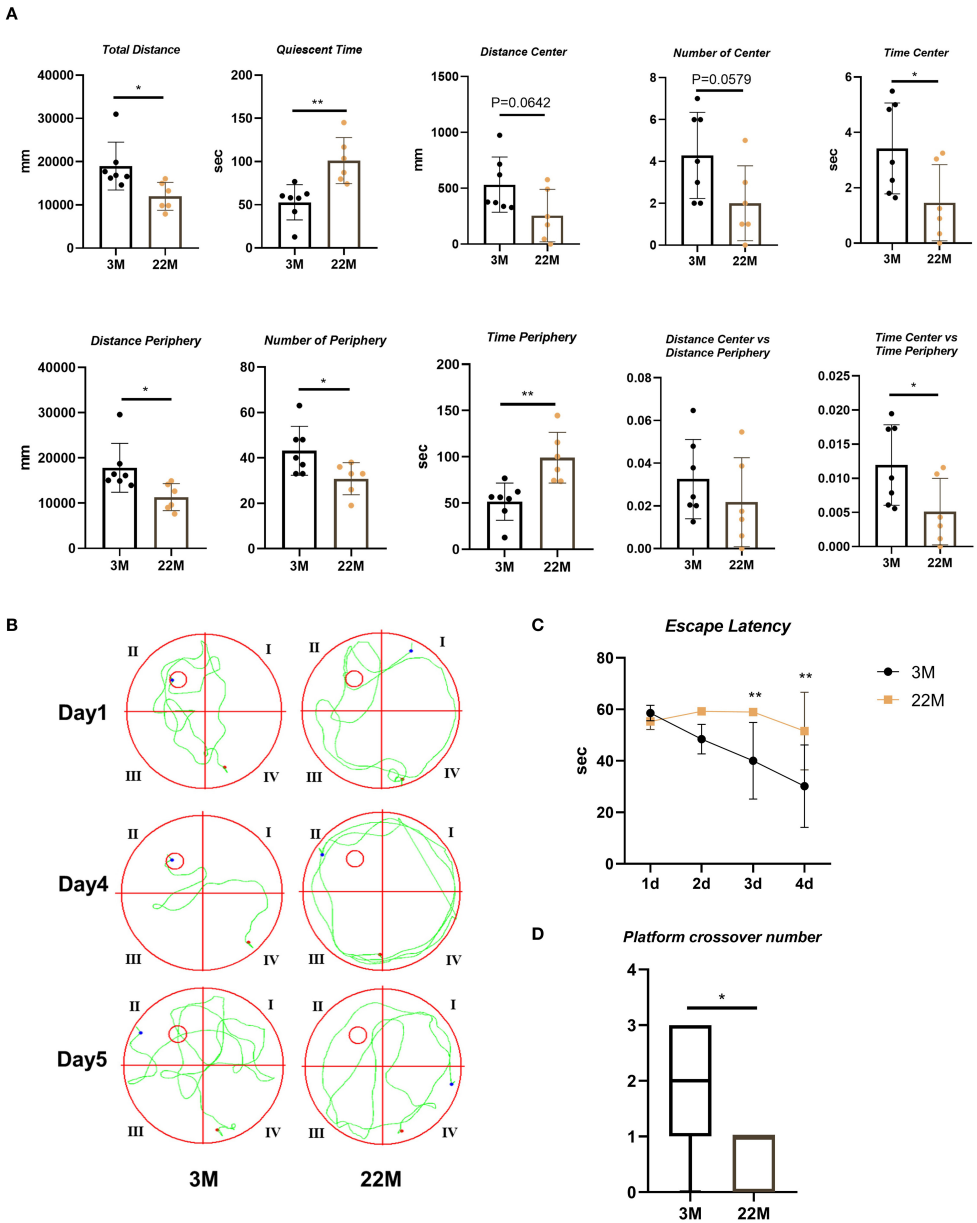
Evaluation of exploration and spatial memory ability in aging mice. **(A)** The parameters of open field test between young (3M) and aging mice (22M) group. Data were represented as mean ± SD and were analyzed by Student *t*-test. ^*^P < 0.05 and ^**^P < 0.01. **(B)** The locus diagrams of Morris water maze. Day 1th to Day 4th, Navigation test; Day 5th, Space exploration test. **(C)** Escape Latency in the navigation test of Morris water maze. Data were represented as mean ± SD and were analyzed by two-way analysis of variance (ANOVA) following Bonferroni's multiple comparisons test. ^**^P < 0.01. **(D)** Platform crossover number in the space exploration test of Morris water maze. Mann-Whitney *U* test was employed and a box plot was used. ^*^P < 0.05.

To evaluate the exploration ability and spatial memory ability of aging mice, we normally raised 3-month-old mice (young) and 22-month-old mice (aged), and evaluated behavioral memory using the open field test and Morris water maze. In the open field test, aging mice displayed impaired exploration ability, including decreased Total Distance (*P*<0.05), Time Center (*P*<0.05), Distance Periphery (*P*<0.05), Number of Periphery (*P*<0.05), and Time Center vs Time Periphery (*P*<0.05), while increased Quiescent Time (*P*<0.01) and Time Periphery (*P*<0.01); and a slight downward trend in Distance Center, Number of Center and Distance Center vs. Distance Periphery but no statistical differences (Figure 1A). Morris water maze were used to evaluate the hippocampus-dependent spatial memory. In Morris Water Maze Test, aging mice witnessed extended escape latency in the navigation test (Two-way ANOVA, *P*<0.01 in 3d and 4d) and reduced platform crossover number in the space exploration test (Mann-Whitney U test, *P*< 0.05), compared with young mice (Figures 1B–D). This showed that the exploratory ability and spatial memory ability of aging mice decreased with age.

### Aging mice showed degeneration and aging of hippocampal neurons

**Figure 2:**
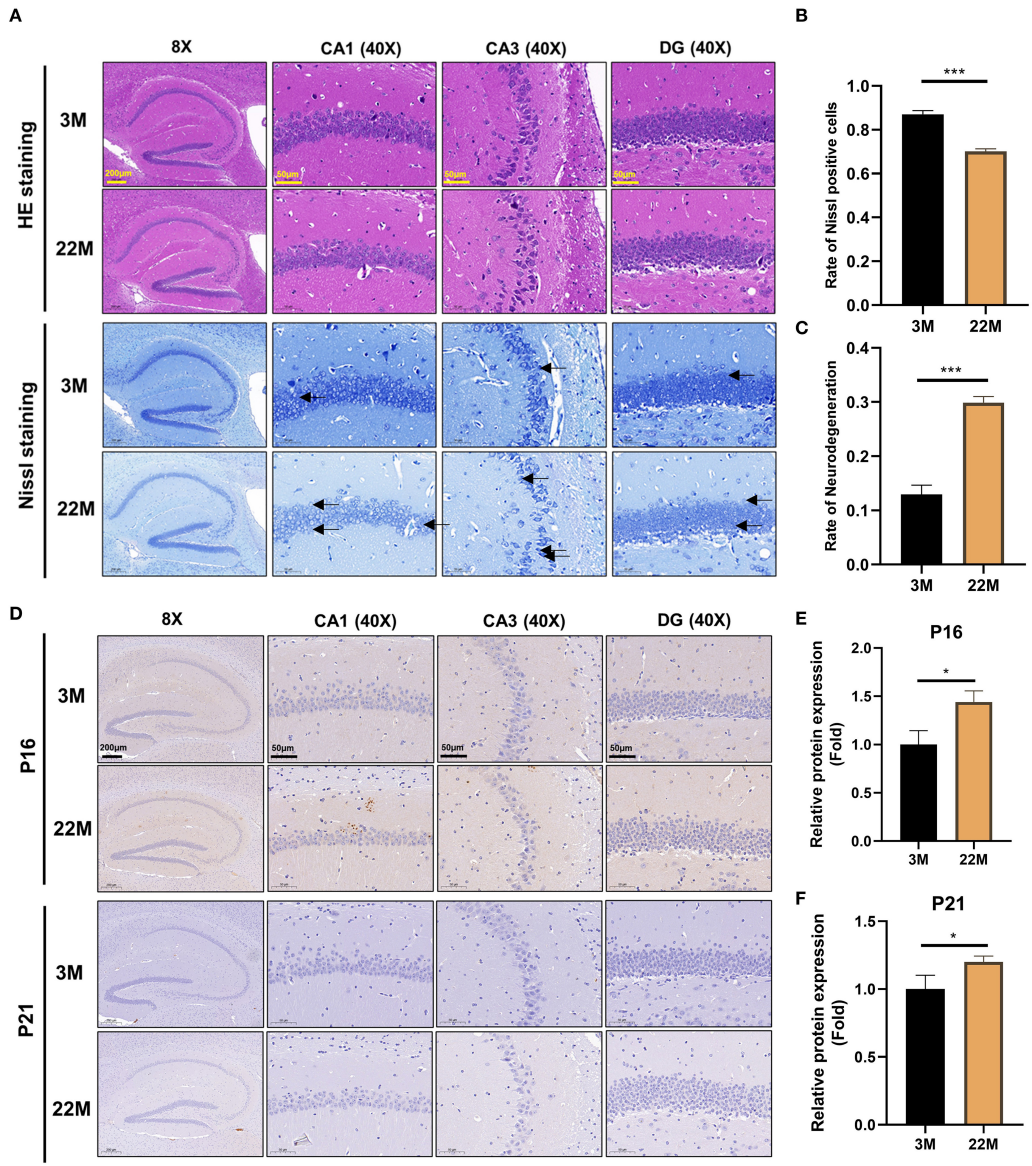
Evaluation of hippocampal histomorphology in aging mice. **(A)** H&E and Nissl staining in the hippocampus between 3M and 22M group. The magnifications and scale bars were shown in **(A)**. **(B,C)** Nissl positive cells and Neurodegeneration rate in the hippocampus were assessed by Nissl staining. Data were represented as mean ± SD and were analyzed by Student *t*-test. ***P < 0.001. Arrows showed degenerated neurons. **(D)** IHC of senescence markers P16 and P21 in the hippocampus. The magnifications and scale bars were shown in **(D)**. **(E,F)** Relative expressions of senescence markers P16 and P21 were calculated by IHC. Data were represented as mean ± SD and were analyzed by Student *t*-test. *P < 0.05.

To more intuitively reflect the pathological manifestations of the hippocampal neurons of aging mice, the CA1, CA3, and DG regions of hippocampus were assessed by HE staining and Nissl staining (Figure 2A). The nerve cells of the young group had intact cell structure, clear round shape, and were arranged compactly and neatly; while the nerve cells of aging mice in hippocampus were disordered and the granules of Nissl bodies were blurred (Figure 2A). Accordingly, quantitative analysis revealed that the number of Nissl bodies decreased (*P*<0.001), while the rate of neurodegeneration increased significantly (*P*<0.001) in the hippocampal neurons of aging mice than that of young mice (Figures 2B,C). The expressions of senescence markers p21 and p16 protein detected by IHC in aging murine hippocampal neurons was higher (*P*<0.05) than those of young mice, implying that aging murine hippocampus showed the characteristics of aging (Figures 2D–F).

### mRNA-seq and IHC of hippocampus indicated the imbalance of GABRB2 and GABRA2 expression in aging mice

**Figure 3:**
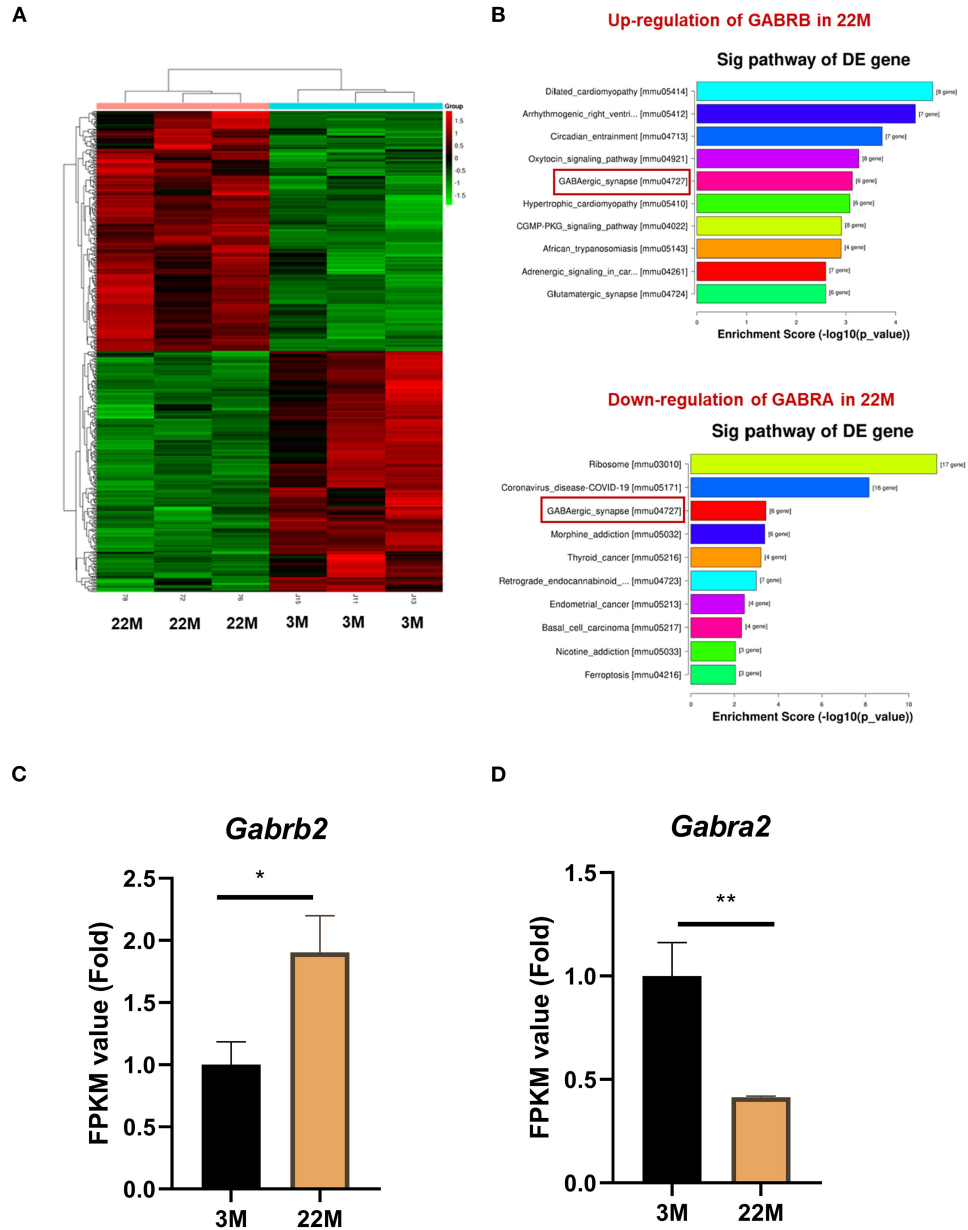
GABRB2 and GABRA2 expression were evaluated by mRNA-seq of hippocampus in aging mice. **(A)** Heat map of hippocampus between 3M and 22M group. **(B)** KEGG analysis of the hippocampus between 3M and 22M group. **(C,D)** FPKM value of GABRB2 and GABRA2 in the hippocampus by mRNA-seq. Data were represented as mean ± SD and were analyzed by Student *t*-test. *P < 0.05 and **P < 0.01.

**Figure 4:**
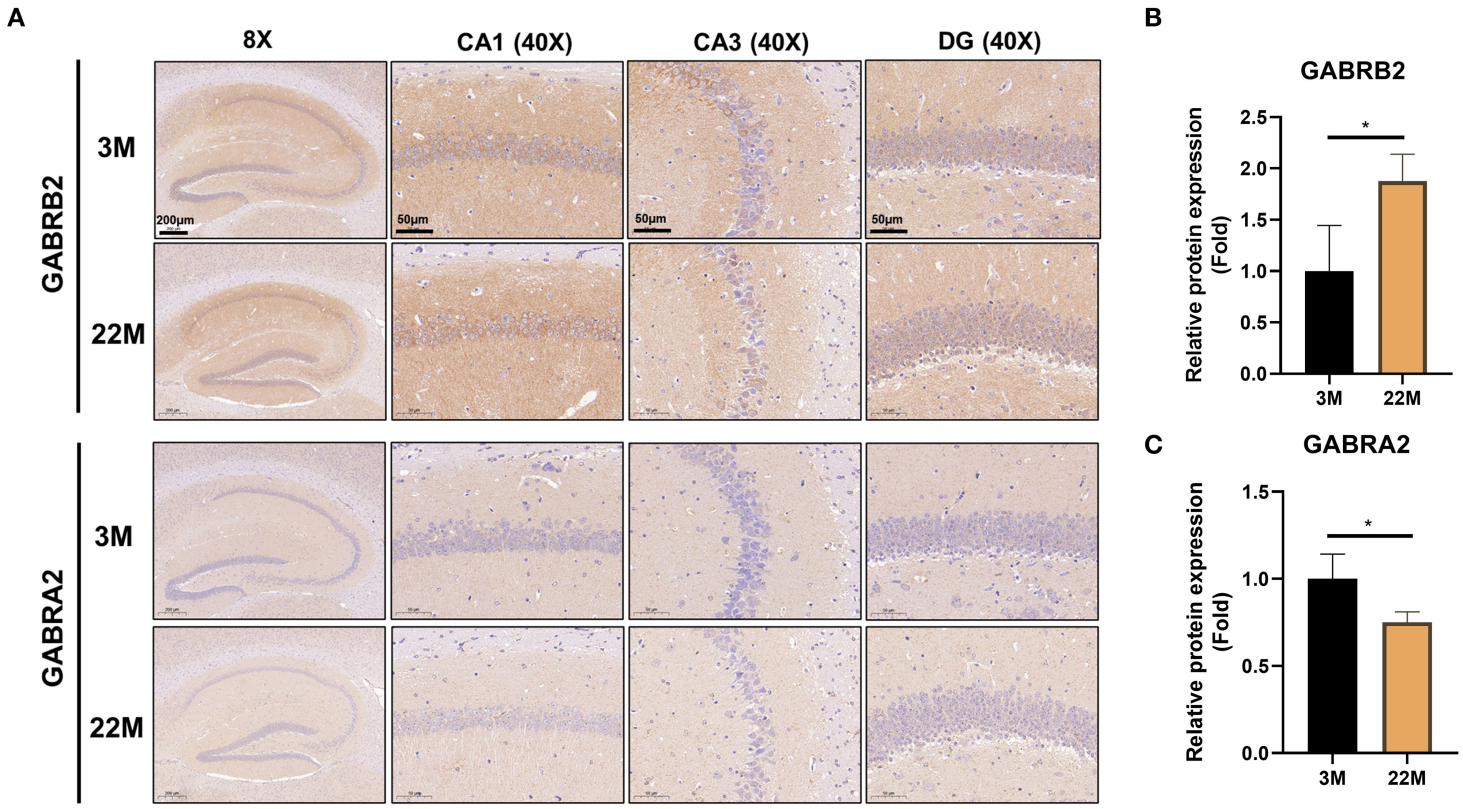
Validation of GABRB2 and GABRA2 expression by IHC of hippocampus in aging mice. **(A)** IHC of GABRB2 and GABRA2 in the hippocampus between 3M and 22M group. The magnifications and scale bars were shown in **(A)**. **(B,C)** Relative expressions of GABRB2 and GABRA2 were calculated by IHC. Data were represented as mean ± SD and were analyzed by Student *t*-test. *P < 0.05.

To further explore the mechanism of hippocampus aging in mice, we employed mRNA-seq to screen for possible genes and pathways in the young (3M) and aging (22M) hippocampus. The heat map displayed up-regulated and down-regulated gene sets (Figure 3A). KEGG pathway enrichment analysis found that GABAergic synapse was involved in both up-regulated and down-regulated signaling pathways (Figure 3B). We further analyzed the differential expression genes and found that GABRB2 (*P*<0.05) was up-regulated in the aging hippocampus whereas GABRA2 (*P*<0.01) was down-regulated in the aging hippocampus, indicating an imbalance of GABRB2 and GABRA2 expressions in aging mice (Figures 3C,D). We then employed hippocampal immunohistochemistry to further validate the GABRB2 and GABRA2 protein expressions in aging mice. Consistent with the mRNA-seq results, we verified that GABRB2 protein expression (*P*<0.01) was increased in the aging hippocampus whereas GABRA2 protein expression (*P*<0.01) was decreased in the aging hippocampus (Figures 4A–C). This study laid a foundation for further study on the mechanism of MA in improving hippocampus aging.

### MA alleviated the damage of exploration and spatial memory ability in aging mice

**Figure 5:**
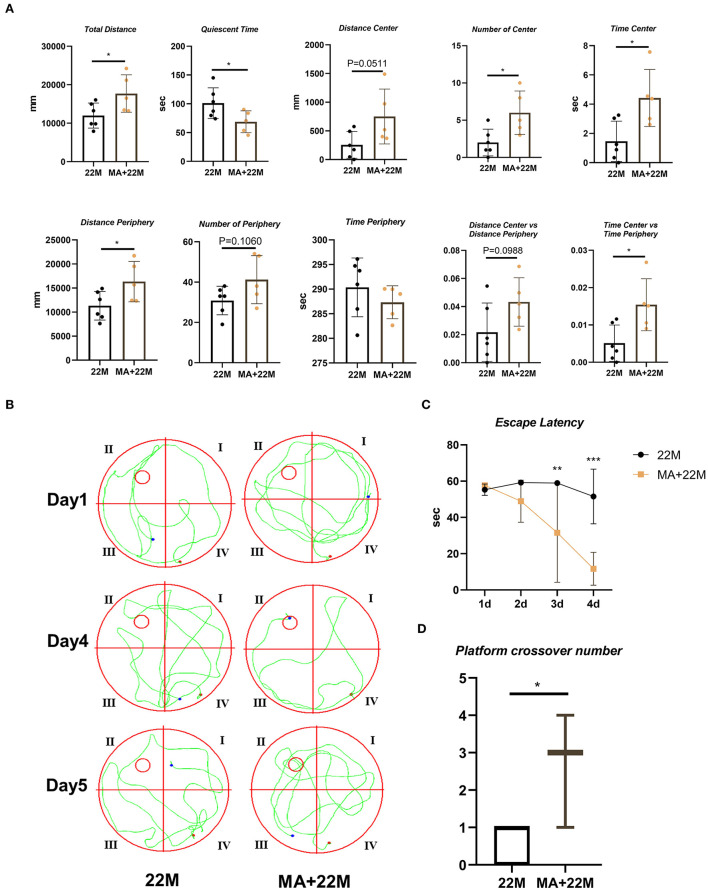
Evaluation of exploration and spatial memory ability in MA-treated aging mice. **(A)** The parameters of open field test between aging mice (22M) and MA-treated aging mice (MA+22M) group. Data were represented as mean ± SD and were analyzed by Student *t*-test. *P < 0.05. **(B)** The locus diagrams of Morris water maze between 22M and MA+22M group. Day 1th and Day 4th, Navigation test; Day 5th, Space exploration test. **(C)** Escape Latency in the navigation test of Morris water maze between 22M and MA+22M group. Data were represented as mean ± SD and were analyzed by two-way ANOVA following Bonferroni's multiple comparisons test. **P < 0.01 and ***P < 0.001. **(D)** Platform crossover number in the space exploration test of Morris water maze. Mann-Whitney *U* test was employed and a box plot was used. *P < 0.05.

MA, a saturated fatty acid, was a promising anti-aging effective constituent in previous studies. First, we evaluated the safety of MA treatment on aging mice. Using HE staining, we found that MA could not lead to damage to organs, including heart, liver, spleen, lung, kidney, and testis (Supplementary Figure 1). Expectedly, MA alleviated hippocampal aging to some extent. In the open field test, we examined an improved exploration ability in aging mice, including increased Total Distance (*P* < 0.05), Number of Center (*P*<0.05), Time Center (*P*<0.05), and Time Center vs Time Periphery (*P*<0.05); and decreased Quiescent Time (*P*<0.05); and an upward trend in Distance center and Distance Center vs Distance Periphery and a downward trend in Time Periphery (Figure 5A). In Morris water maze, we observed a reduced escape latency in the navigation test (Two-way ANOVA, *P*<0.01 in 3d and *P*<0.001 in 4d) and increased platform crossover number (Mann-Whitney U test, *P*<0.05) in the space exploration test, indicating MA improved hippocampus-dependent spatial memory (Figures 5B–D).

### MA ameliorated degeneration and aging of hippocampal neurons

**Figure 6:**
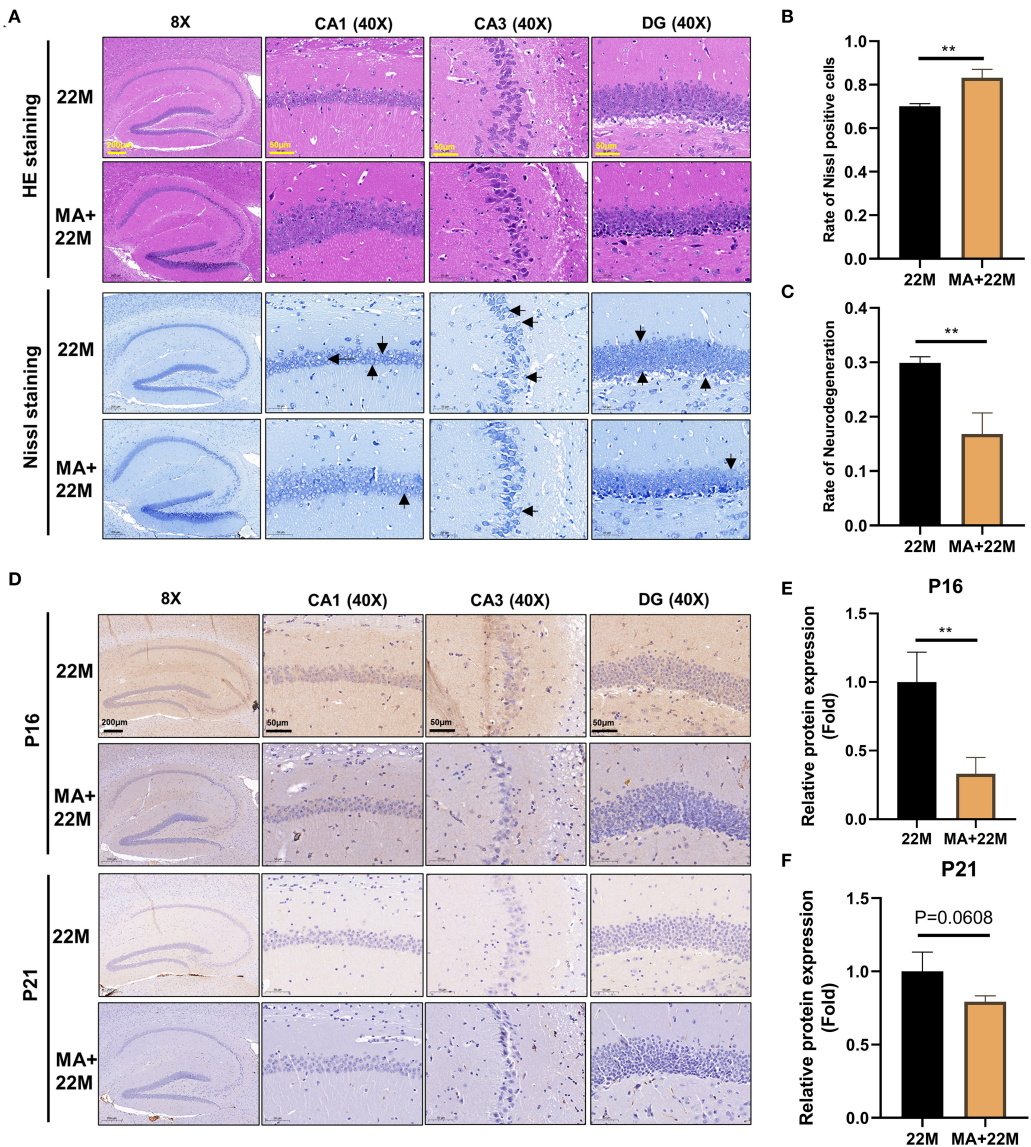
Evaluation of hippocampal histomorphology in MA-treated aging mice. **(A)** H&E and Nissl staining in hippocampus between 22M and MA+22M group. The magnifications and scale bars were shown in **(A)**. **(B,C)** Nissl positive cells and Neurodegeneration percentage in hippocampus were assessed by Nissl staining between 22M and MA+22M group. Data were represented as mean ± SD and were analyzed by Student *t*-test. **P < 0.01. Arrows showed degenerated neurons. **(D)** IHC of senescence markers P16 and P21 in the hippocampus between 22M and MA+22M group. The magnifications and scale bars were shown in **(D)**. **(E,F)** Relative expressions of senescence markers P16 and P21 calculated by IHC. Data were represented as mean ± SD and were analyzed by Student *t*-test. **P < 0.01.

We further used H&E and Nissl staining to demonstrate the effect of MA on hippocampal histomorphology. We found an improvement in the disorder of hippocampal neurons and clearer Nissl bodies in MA-treated aging mice (Figure 6A). Accordingly, our quantitative results also showed that the rate of Nissl positive cells was increased (*P*<0.01) and the rate of neurodegeneration was decreased (*P*<0.01) (Figures 6B,C). Furthermore, hippocampal immunofluorescence of nerve regeneration marker Nestin demonstrated that MA increased Nestin protein expression (*P*<0.05); an increased trend of Sox2, another nerve regeneration marker, was observed in MA-treated aging hippocampus (Supplementary Figures 2A–D). IHC of Classic aging marker P16 also displayed that MA-treated aging hippocampus reduced the aging marker P16 protein expression (*P*<0.01). There was a decreasing trend of P21 protein expression in the aging hippocampus, although there was no statistical difference (Figures 6D–F). We also explored the effect of MA on cerebral cortex neurons and found that MA could alleviate neurodegeneration of cerebral cortex and reduce aging marker P16 expression (Supplementary Figures 3A–D).

### MA alleviated hippocampal aging correlated with GABAergic signaling

**Figure 7:**
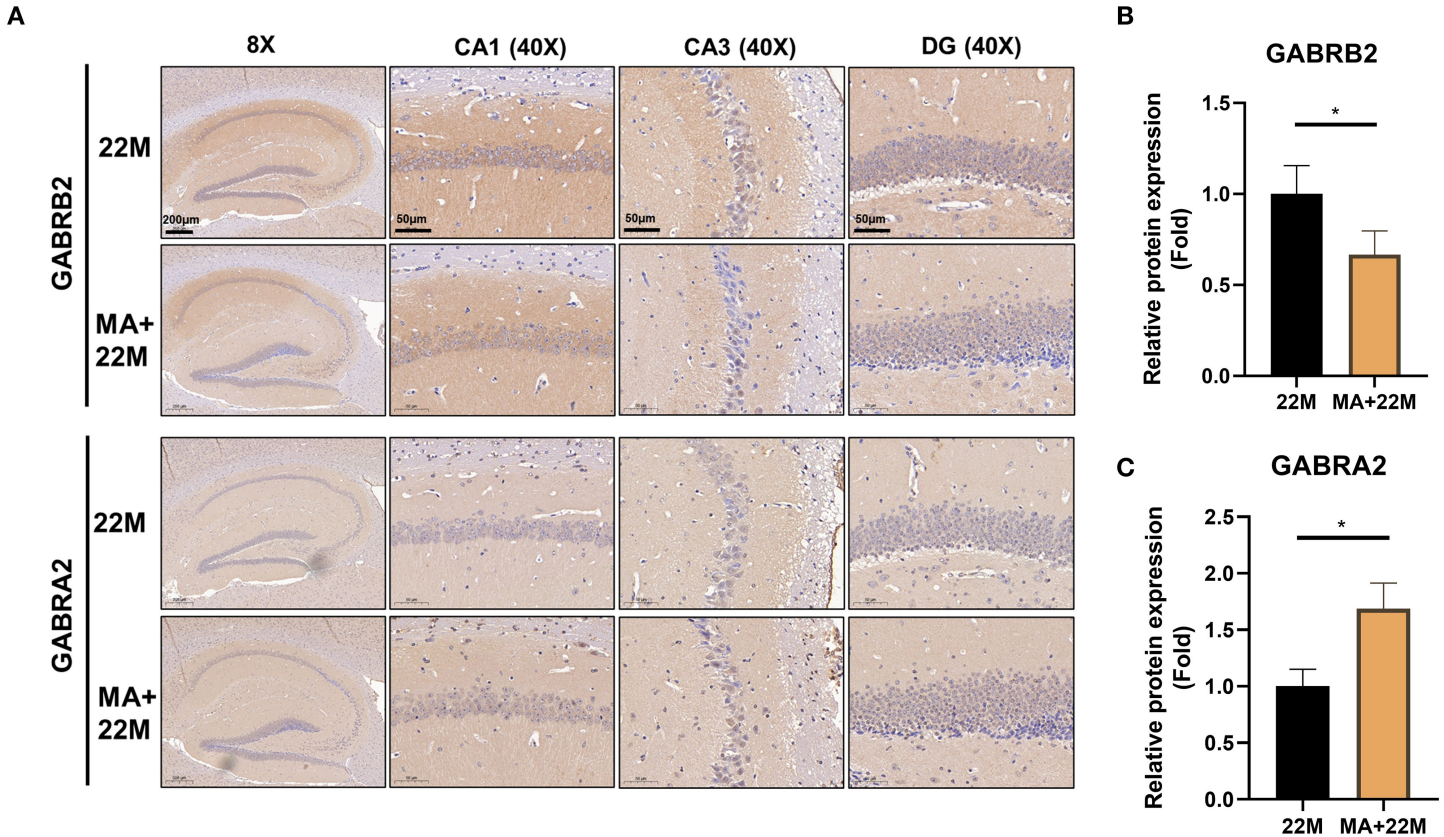
Validation of GABRB2 and GABRA2 expression by IHC of hippocampus in MA-treated aging mice. **(A)** IHC of GABRB2 and GABRA2 in the hippocampus between 22M and MA+22M group. Magnifications and scale bars were shown in **(A)**. **(B,C)** Relative expressions of GABRB2 and GABRA2 were calculated by IHC. Data were represented as mean ± SD and were analyzed by Student *t*-test. *P < 0.05.

To further explore the mechanism of MA-treated hippocampus aging in mice, we employed hippocampus IHC to further validate the GABRB2 and GABRA2 protein expressions in MA-treated aging mice. We verified that GABRB2 protein expression was decreased (*P*<0.05) in the aging hippocampus whereas GABRA2 protein expression was increased (*P*<0.05) in MA-treated aging hippocampus, indicating that MA may improve the imbalance of GABAergic expressions (Figures 7A–C, Supplementary Figures 4A–D).

## Discussion

Our study demonstrated that MA, a saturated fatty acid extracted from cardamom oil, coconut oil, palm kernel oil, etc., ameliorated the aging of hippocampus correlated with GABAergic signaling. Aging mice displayed a decline in exploration and spatial memory ability, impairment in the hippocampal histomorphology, upregulation of GABRB2 and downregulation of GABRA2 expression; these effects were improved by the administration of MA.

In aging mice of 22M, a decreased exploration and spatial memory ability were observed using open field test and Morris water maze, which were consistent with previous studies (20, 21). As we know, exploration and spatial memory ability assessed by open field test and Morris water maze respectively are hippocampus-dependent type of exploration and memory ability (22, 23). Hippocampus, as an important part of the limbic system of the brain, is a high-level regulatory center of the neuroendocrine system, whose function is closely related to aging. Aging is characterized by a decrease of hippocampal neurons (5), which was verified by H&E and Nissl staining in our study. To further explore the mechanism of hippocampus aging, we employed mRNA-seq to detect differential gene expressions in the hippocampus between young and aging mice. We found that GABAergic synapse, oxytocin and endocannabinoid signaling were involved in this process. Aging induced oxytocinergic transmission impairment in the central nervous system (24); oxytocin in people aged over 65 may be associated with aging-related changes in hippocampus (25). Furthermore, oxytocin induced GABA release onto mossy cells of the rat dentate gyrus of hippocampus (26). Endocannabinoid system activity contributes to the homeostatic defense against aging and thus may retard brain aging (27, 28). Besides, cannabinoid type 1 receptor activity on hippocampal GABAergic neurons ameliorated age-dependent cognitive decline by decreasing pyramidal cell degeneration and neuroinflammation (29). Given that both oxytocin and endocannabinoid signaling can participate in the regulation of the GABA pathway, we focused on GABAergic signaling in this study. KEGG pathway analysis was found that GABRB2 was upregulated whereas GABRA2 was downregulated, which was verified by IHC of hippocampus between young and aging mice. Previous studies also demonstrated that GABRA2 expression decreased with age in the hippocampus (9, 10). However, aging-related disorders on GABRB2 expression are contradictory. The mRNA level of GABRB2 in the nucleus accumbens and prefrontal cortex was decreased in middle-aged female mice (30). However, kainic acid, an epileptic seizures inducer in mice, could downregulate GABRB2 expression (31). The reasons of contradictory results of GABRB2 expression may be due to the differences in brain regions and disease detections. Overall, we implied an imbalance of GABAergic signaling in the hippocampus between young and aging mice, which may provide insight into the discovery of targets in aging-related disorders.

To further explore the anti-aging effect of drugs through the GABAergic signaling pathway, MA, a saturated fatty acid, was selected and employed to verify the effect and mechanism of anti-hippocampus aging. Studies have reported that aging was accompanied by a decline in MA, indicating MA played an important role in aging-related disorders (14). However, contradicting our study, Zeng et al. pointed out that MA may promote tumor progression and drug resistance, which may be due to different diseases studied and different drug doses (32). Furthermore, previous studies have demonstrated that MA could promote the proliferation and differentiation of neural stem cells *in vitro* (13). The isomer of MA, Myristoleic acid, could suppress osteoclastogenesis and bone resorption by inhibiting RANKL activation, indicating MA had a potential therapeutic effect on aging-related diseases (15). However, the effect of MA on hippocampal neurons aging has not been reported *in vivo*. We employed 22-month-old naturally aged C57BL/6 mice to evaluate the effect and mechanism of MA on hippocampal aging. Our investigations indicated that MA alleviated hippocampal aging correlated with GABAergic signaling. To the best of our knowledge, this is the first time that we report that MA improved hippocampal aging, which was expected to provide new drugs for combating aging-related diseases.

Our current study has some limitations. We performed *in vivo* study to verify the effect of MA alleviating hippocampus aging via regulating GABAergic expressions. However, there is still a lack of *in vitro* experiments to further explore the effect and mechanism of MA in improving the aging of hippocampal neurons. Secondly, the knockout and overexpression of GABRA2 and GABRB2 in animals and hippocampal neurons are worthy of further exploration to verify the role and mechanism of hippocampal aging and MA in improving hippocampal neuronal aging. As a potential nutritional supplement for the treatment of aging-related diseases, such as Alzheimer's disease and osteoporosis, MA is worthy of further verification by clinical trials.

**Figure 8:**
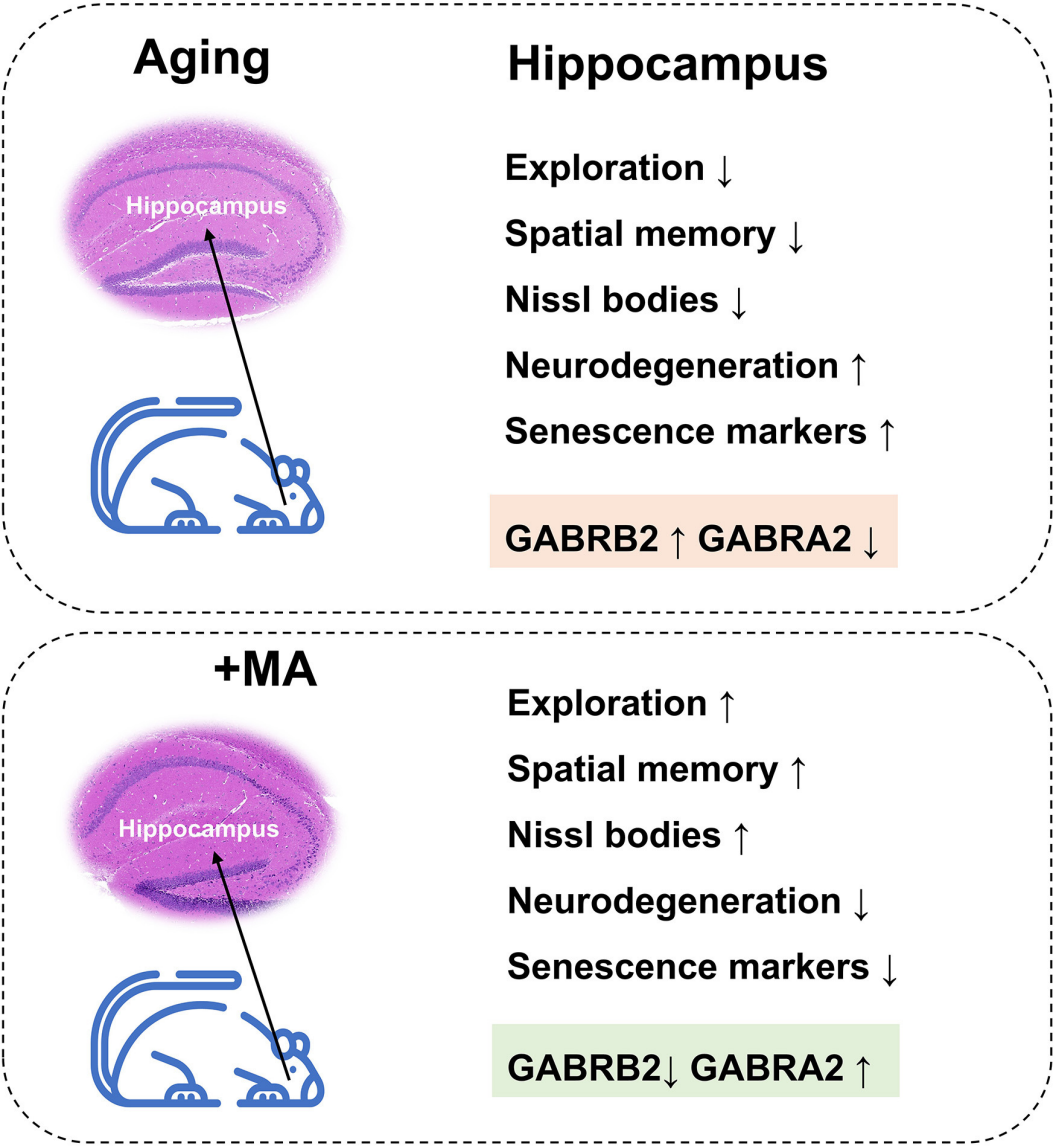
Working model. Aging mice displayed a decline in exploration and spatial memory ability, impairment in hippocampal histomorphology, upregulation of GABRB2, and downregulation of GABRA2 expression; these effects were improved by the administration of MA.

In conclusion, we demonstrated a decline in exploration and spatial memory ability and impairment of hippocampal histomorphology in aging mice, which may result from an imbalance of GABRB2 and GABRA2 expressions. MA, a saturated fatty acid extracted from food, alleviated the aging of hippocampus correlated with GABAergic signaling (Figure 8). Our study indicated that MA has the potential as a nutritional supplement, which may provide insight into the treatment and prevention of aging-associated diseases.

## Data availability statement

The datasets presented in this study can be found in online repositories. The names of the repository/repositories and accession number(s) can be found below: NCBI; GSE201029.

## Ethics statement

The animal study was reviewed and approved by the First Affiliated Hospital of Guangzhou University of Chinese Medicine.

## Author contributions

QS, XJ, HR, and GS designed the project. QS, GC, PZ, WZ, HC, DY, FY, HL, XZ, and JH performed the experiments. QS, GC, and PZ wrote the original draft of the manuscript. XJ, HR, GS, and DL reviewed and edited the manuscript, supervised the research, and all authors analyzed the data. All authors read and approved the final manuscript.

## Funding

This work was supported in part by the following grants: National Natural Science Foundation of China (81904225), Guangzhou Science and Technology Project (202201011169), Guangdong Natural Science Foundation (2020A1515110322 and 2021A1515011247 and 2022A1515012062), Innovative Team Project of the Department of Education of Guangdong Province (2021KCXTD017), High-Level University Collaborative Innovation Team of GZUCM (2021xk57), Graduate Research Innovation Project of GZUCM (A1-2606-21-429-001Z22). The funding institutions had not any role in the study design, data collection, data analysis, interpretation, or writing of the report in this study.

## Conflict of interest

The authors declare that the research was conducted in the absence of any commercial or financial relationships that could be construed as a potential conflict of interest.

## Publisher's note

All claims expressed in this article are solely those of the authors and do not necessarily represent those of their affiliated organizations, or those of the publisher, the editors and the reviewers. Any product that may be evaluated in this article, or claim that may be made by its manufacturer, is not guaranteed or endorsed by the publisher.
