# Supplementary material for: Myristic acid alleviates hippocampal aging correlated with GABAergic signaling
Source: Front Nutr. 2022 Sep 8;9:907526. doi: 10.3389/fnut.2022.907526 (PMC9493098; doi:10.3389/fnut.2022.907526)
Supplement: Supplementary file 1 [file Data_Sheet_1.PDF]

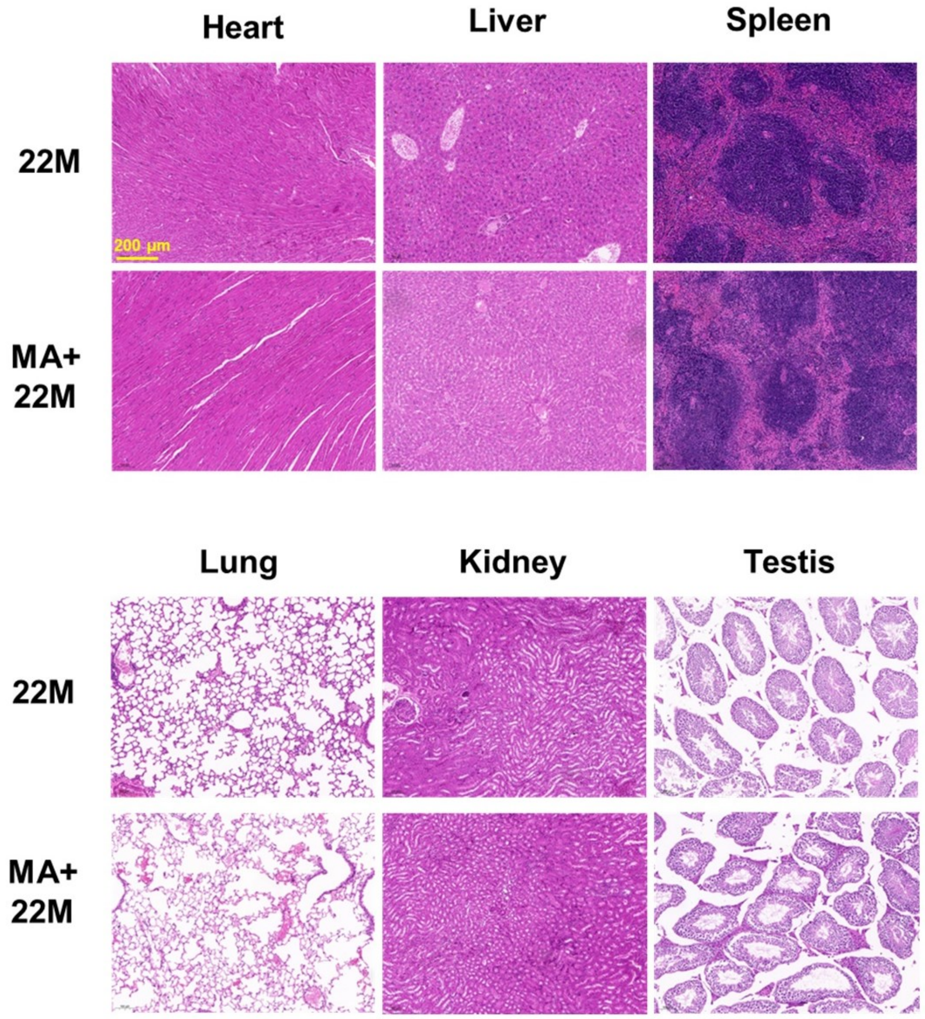

Supplementary Figure 1. Safety of MA treating aging mice on the organs including heart, liver, spleen, lung, kidney, and testis by HE staining.

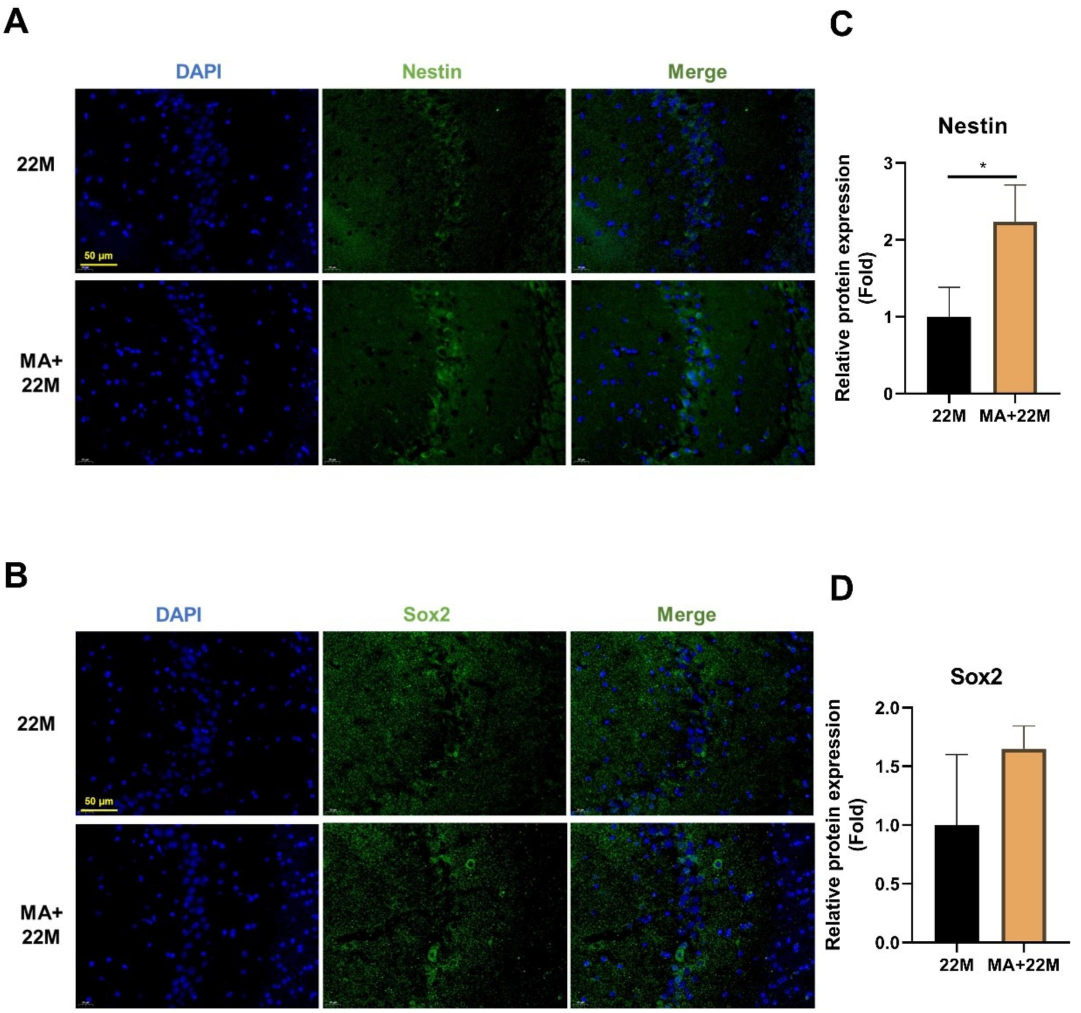

Supplementary Figure 2. Hippocampal Nestin and Sox2 were detected by immunofluorescence (IF) in MA-treated aging mice. (A-B) IF of Nestin and Sox2 in the hippocampus between 22M and MA+22M group. Magnifications: 40×; Scale bars: 50 μm. **(C-D)** Relative expressions of Nestin and Sox2 were calculated by IF. Data were represented as mean ± SD and were analyzed by Student *t*-test. *P < 0.05.

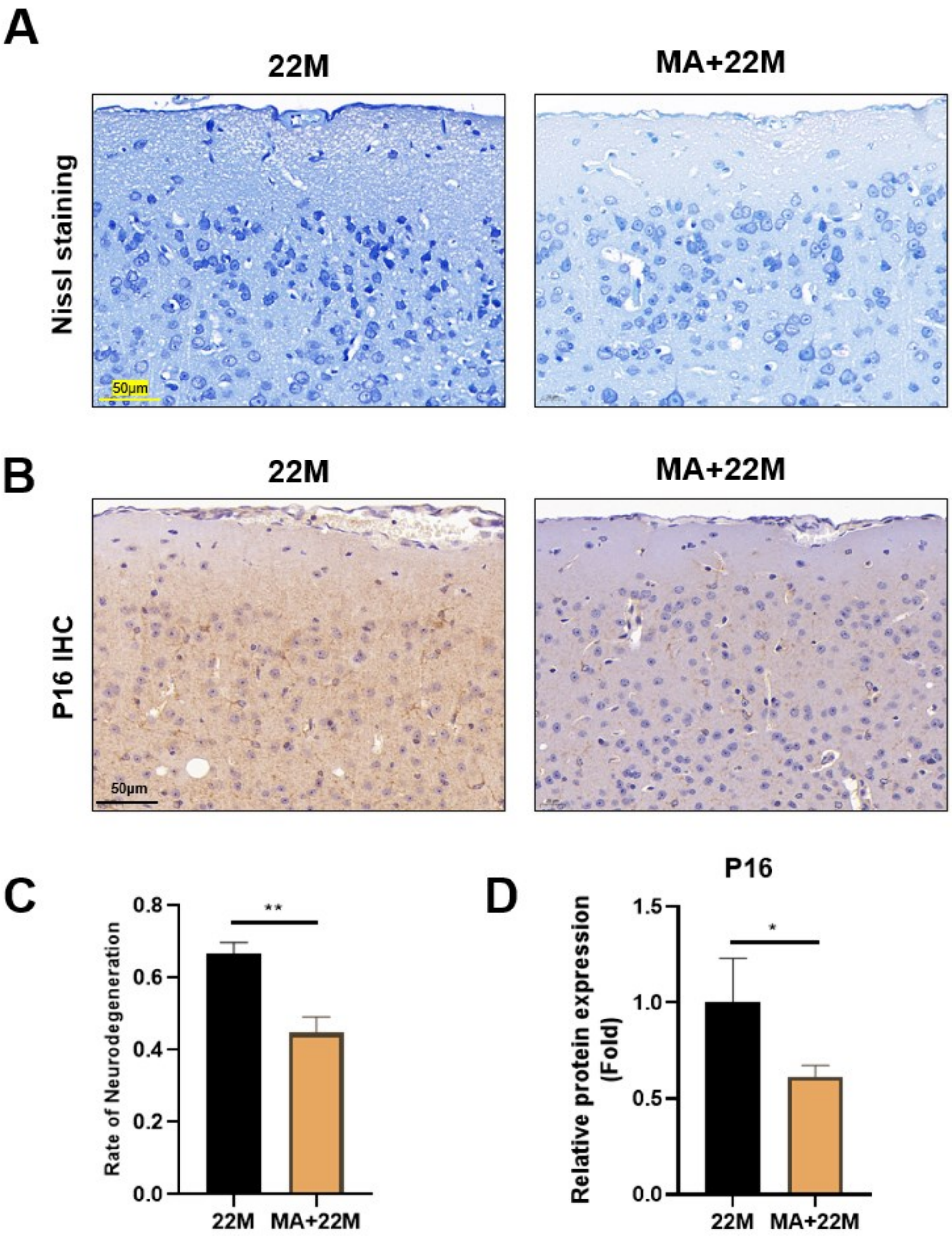

Supplementary Figure 3. Evaluation of cortex histomorphology in MA-treated aging mice. (A) Representative images of Nissl staining in the cortex. Magnifications: 40×; Scale bars: 50 μm. **(B)** Representative images of P16 IHC in the cortex. Magnifications: 40×; Scale bars: 50 μm. **(C)** The statistics of the rate of neurodegeneration in the cortex. Data were represented as mean ± SD and were analyzed by Student *t*-test. $^{**}P < 0.01$. **(D)** The statistics of the relative P16 protein expression in the cortex. Data were represented as mean ± SD and were analyzed by Student *t*-test. $^{*}P < 0.05$.

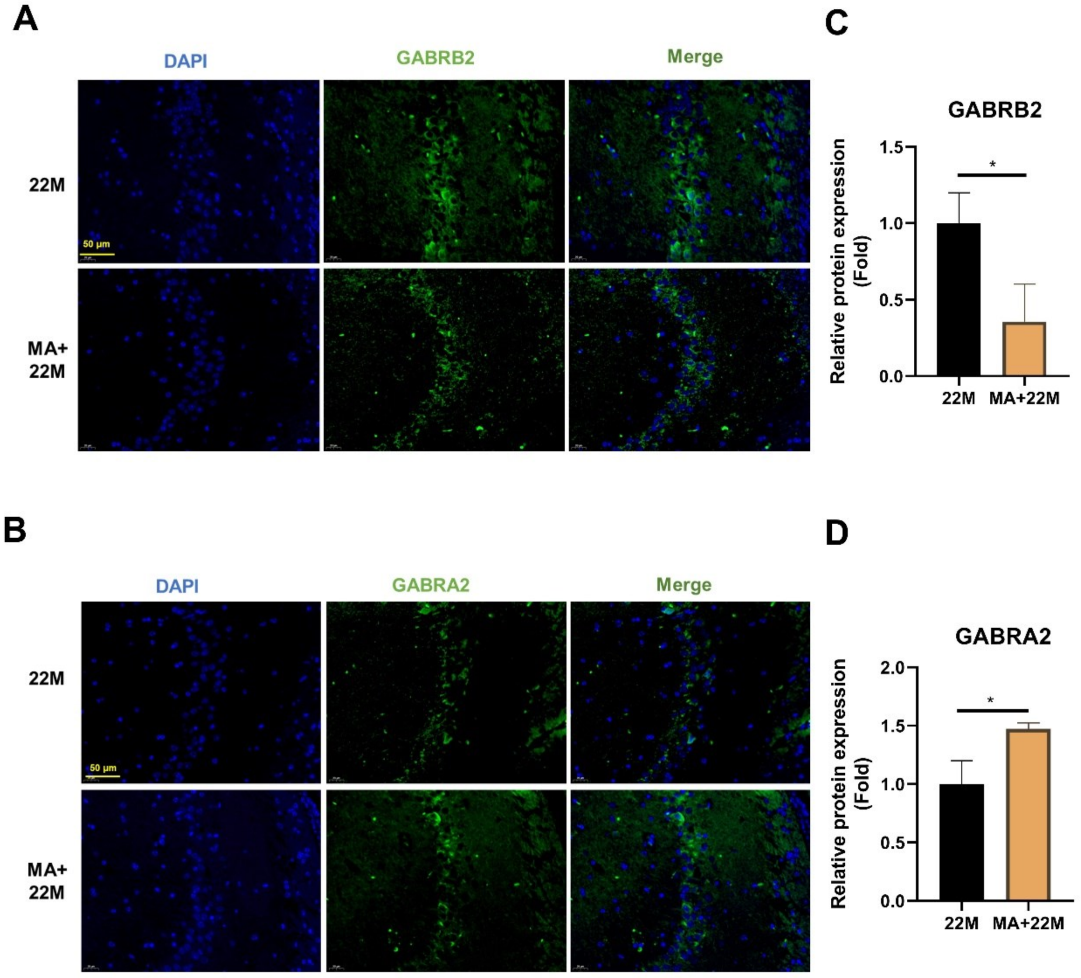

Supplementary Figure 4. Hippocampal GABRB2 and GABRA2 were detected by IF in MA-treated aging mice. (A-B) IF of GABRB2 and GABRA2 in the hippocampus between 22M and MA+22M group. Magnifications: 40×; Scale bars: 50 μm. **(C-D)** Relative expressions of GABRB2 and GABRA2 were calculated by IF. Data were represented as mean ± SD and were analyzed by Student *t*-test. *$P < 0.05$.
